# Antibodies elicited by altSonflex1-2-3 GMMA vaccine are bactericidal against a panel of drug-resistant *Shigella* clinical isolates

**DOI:** 10.3389/fimmu.2025.1652460

**Published:** 2025-09-04

**Authors:** Elena Boero, Roberta Di Benedetto, Giacomo Vezzani, Renzo Alfini, Pieter-Jan Ceyssens, Giannoula S. Tansarli, Ferric C. Fang, Carla Fontana, Alberto Rossi, Stefania Carrara, Francesca Mancini, Miren Iturriza, Claudia Sala, Carlo Giannelli, Omar Rossi, Francesca Micoli

**Affiliations:** ^1^ GSK Vaccines Institute for Global Health (GVGH), Siena, Italy; ^2^ Division of Human Bacterial Diseases, Sciensano, Brussels, Belgium; ^3^ Department of Laboratory Medicine and Pathology, University of Washington School of Medicine, Seattle, WA, United States; ^4^ Division of Allergy & Infectious Diseases, University of Washington School of Medicine, Seattle, WA, United States; ^5^ Laboratory of Microbiology and Biological Bank, National Institute for Infectious Diseases Lazzaro Spallanzani IRCCS, Rome, Italy; ^6^ Monoclonal Antibody Discovery Laboratory, Fondazione Toscana Life Sciences, Siena, Italy

**Keywords:** Shigella, altSonflex1-2-3, vaccine, AMR, O-antigen, SBA, GMMA

## Abstract

Shigellosis significantly impacts global health, particularly affecting vulnerable populations in low- and middle-income countries, with over 270 million annual infections. It also causes morbidity in specific high-risk groups in high-income countries. Antibiotic treatment is increasingly compromised by multidrug-resistant strains, highlighting the urgent need for a *Shigella* vaccine. We developed a four-component O-antigen-based vaccine candidate targeting *Shigella sonnei* and *Shigella flexneri* 1b, 2a, and 3a serotypes, named altSonflex1-2-3, to provide broad protection against most globally prevalent *Shigella* strains. Here, we characterized the O-antigen structural features of a panel of *S. flexneri* drug-resistant clinical isolates and verified that they did not significantly differ from the O-antigen in the vaccine. Preclinical sera elicited by altSonflex1-2–3 were bactericidal against most strains, confirming the ability of anti-O-antigen antibodies to recognize and kill *in vitro* different clinical isolates. Importantly, our results suggest that altSonflex1-2–3 could offer protection against antimicrobial-resistant *Shigella* strains, addressing a critical public health issue.

## Graphical Abstract

The artwork used in this figure was adapted from Servier Medical Art (http://https://smart.servier.com/). Servier Medical Art by Servier is licensed under a CC BY 4.0 License.

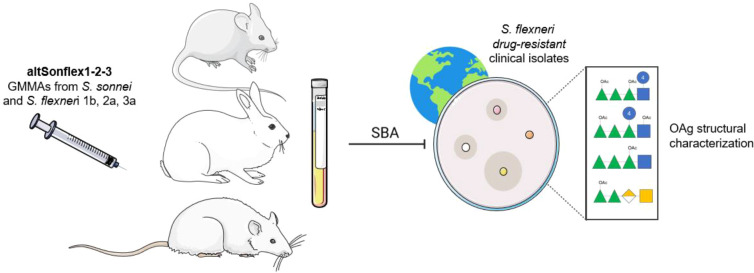

## Introduction

*Shigella* spp. are the etiological agents of shigellosis, a diarrheal disease caused by bacterial infection of the colonic epithelium through oral–fecal transmission (1–3). Shigellosis is the second leading cause of enteric disease mortality worldwide after rotavirus disease, with over 270 million infections and 148,000 fatalities annually, predominantly affecting children under 5 years of age and individuals over 70 years old in low- and middle-income countries (LMICs) (4–6). Beyond mortality, shigellosis leads to growth retardation, cognitive development issues, and increased susceptibility to other infections (7, 8).

In high-income countries (HICs), shigellosis typically presents as a self-limited illness that resolves without medical intervention. However, severe cases are consistently reported to health authorities each year. The European Centre for Disease Prevention and Control (ECDC) reported 4,149 cases in 2022 across 30 European countries (1.5 cases per 100,000 people). These cases are predominantly concentrated in certain risk groups such as travelers, persons experiencing homelessness, and gay, bisexual, and other men who have sex with men (gbMSM) in which sexually transmitted shigellosis is emerging (9).

Shigellosis can be caused by four species: in order of frequency, *Shigella flexneri* (predominant in LMICs), *Shigella sonnei* (second most frequent in LMICs and first most frequent in HICs), *Shigella dysenteriae* serotype 1 (predominant in pandemics), and *Shigella boydii* (10). Antibiotics are the only available treatment for severe shigellosis to alleviate symptoms, reduce disease duration, and prevent transmission. The WHO treatment recommendations include oral ciprofloxacin (a fluoroquinolone) as the first-choice antibiotic after ampicillin was discontinued due to bacterial resistance, and ceftriaxone (third-generation cephalosporin) or azithromycin as alternatives (11). Worryingly, both *S. flexneri* and *S. sonnei*, the two species most frequently implicated in shigellosis, have developed multidrug-resistant (MDR) profiles, and some of these species have been implicated in recent shigellosis outbreaks (9, 12–14). Fluoroquinolone-resistant *Shigella* species have been prioritized as high-risk pathogens in the WHO Bacterial Priority Pathogens List 2024 update (15).

Vaccines are crucial in mitigating antimicrobial resistance (AMR) by preventing infections and thus reducing antibiotic use (16–18). Although a commercial vaccine for shigellosis is not yet available, candidates are under development, many including the O-antigen (OAg) component of the lipopolysaccharide, an established protective antigen (19). However, considering the structural diversity of OAg across *Shigella* species and serotypes, a multivalent vaccine approach is necessary to increase coverage (19–22). While *S. sonnei* has a unique OAg structure, all *S. flexneri* serotypes, except *S. flexneri* 6 (23), share the following common OAg tetrasaccharide backbone (corresponding to serotype Y) decorated with glucosylation and *O*-acetylation in different positions that confer serotype specificity (24).

(→2)-α-l-Rha*p*III-(1→2)-α-l-Rha*p*II-(1→3)-α-l-Rha*p*I-(1→3)-β-d-Glc*p*NAc-(1→).

Structural similarities among *S. flexneri* serotypes have been shown to induce cross-reactive immune responses, but the structural features driving immune cross-reactivity remain unclear (1, 25).

A multivalent OAg-based *Shigella* vaccine [named altSonflex1-2-3 (26)] is progressing into Phase 2 trials. This vaccine contains Generalized Modules for Membrane Antigens (GMMA), engineered outer membrane vesicles of *S. flexneri* 2a, *S. flexneri* 1b, *S. flexneri* 3a, and *S. sonnei*, displaying native OAg. It aims to cover over two-thirds of shigellosis cases in Africa and Asia by inducing an immune response against homologous serotypes, plus potential cross-reactivity against *S. flexneri* serotypes not included in the vaccine (3, 27). We have collected preclinical and clinical evidence that altSonflex1-2–3 induces functional antibodies against a panel of homologous and heterologous laboratory strains (3, 26). However, information on the functionality of sera against real-world circulating strains is lacking.

Here, we present a panel of *S. flexneri* clinical isolates from high-income countries displaying different OAg serotypes and AMR profiles. For each strain, we performed an in-depth characterization of the OAg characteristics, the main antigen addressed by the vaccine-induced immune response, to understand eventual structural variability compared to the OAg displayed by GMMA in the altSonflex1-2–3 vaccine.

Furthermore, we explored the ability of multi-species preclinical sera to induce complement-mediated bactericidal activity against this panel of clinical isolates. Serum bactericidal activity represents a well-established *in vitro* functional assay in the field to complement the assessment of the anti-OAg response (22, 28). Without a correlate of protection established for *Shigella*, bactericidal activity has been suggested as an important measure of the functional activity of vaccine-induced antibodies (29, 30).

We assessed the bactericidal activity of pooled sera of animals vaccinated with altSonflex1-2–3 against these clinical drug-resistant isolates, demonstrating vaccine-induced titers comparable to those observed against the same strains in adult individuals in endemic populations, where natural exposure has been associated with protection (31, 32).

## Results and discussion

### Characterization of the OAg structural features of a panel of *S. flexneri* clinical isolates

We collected 12 *S. flexneri* clinical isolates from various locations between 2017 and 2023. Two international, MSM-related clusters are related to two EpiPulse alerts (**Table 1**). For all strains, we tested antibiotic resistance against ampicillin, ceftriaxone, ciprofloxacin, gentamicin, azithromycin, trimethoprim–sulfamethoxazole, meropenem, tetracycline, and chloramphenicol. We found all strains to be partially or fully resistant to at least one antibiotic, with ampicillin resistance being the most common, as reported in the literature.

**Table 1:** *Shigella flexneri* clinical isolate details and antibiotic resistance profile.

Identification code	Serotype	Isolation year	Isolation location	Risk group	References	Ampicillin	Ceftriaxone	Ciprofloxacin	Gentamycin	Azithromycin	Trimethoprim–sulfamethoxazole	Meropenem	Tetracycline	Chloramphenicol
19096	Y*	2019	Seattle, USA	gbMSM	^11^									
210059	1b*	2021	Seattle, USA	gbMSM	^11^									
I03 (75)	3b*	2009	Italy	–										
6A (18)	3b*	2017	Italy	–										
13B (76)	1b*	2023	Italy	–										
S23BD04932	2a**	2023	Belgium	gbMSM	2023-EIP-0023 (1) International alert									
S23BD05840	2a**	2023	Belgium	gbMSM	2023-EIP-0023 (2) International alert									
S23BD08049	2a**	2023	Belgium	gbMSM	National cluster Flex_1									
S23BD02869	1b**	2023	Belgium	gbMSM	2023-EIP-0023 (3)									
S23BD06925	3b**	2023	Belgium	gbMSM	National cluster Flex_3									
S23BD10088	6**	2023	Belgium	gbMSM	National cluster Flex_4									
S23BD10089	4**	2023	Belgium	gbMSM	National cluster Flex_5									

Identification code identifies strains as per related references or per identifier reported in the database of sources (Lazzaro Spallanzani Hospital or Sciensano). Serotypes are reported as follows: *Unknown serotype, analyzed in this paper by chemical characterization; **Declared serotype, confirmed in this paper by chemical characterization. Information on strains and antibiotic resistance profiles assayed by Kirby–Bauer method according to European Committee on Antimicrobial Susceptibility Testing (EUCAST).

Red = resistant, yellow = intermediate, and white = sensitive. gbMSM, gay, bisexual, and other men who have sex with men.

**Figure 1:**
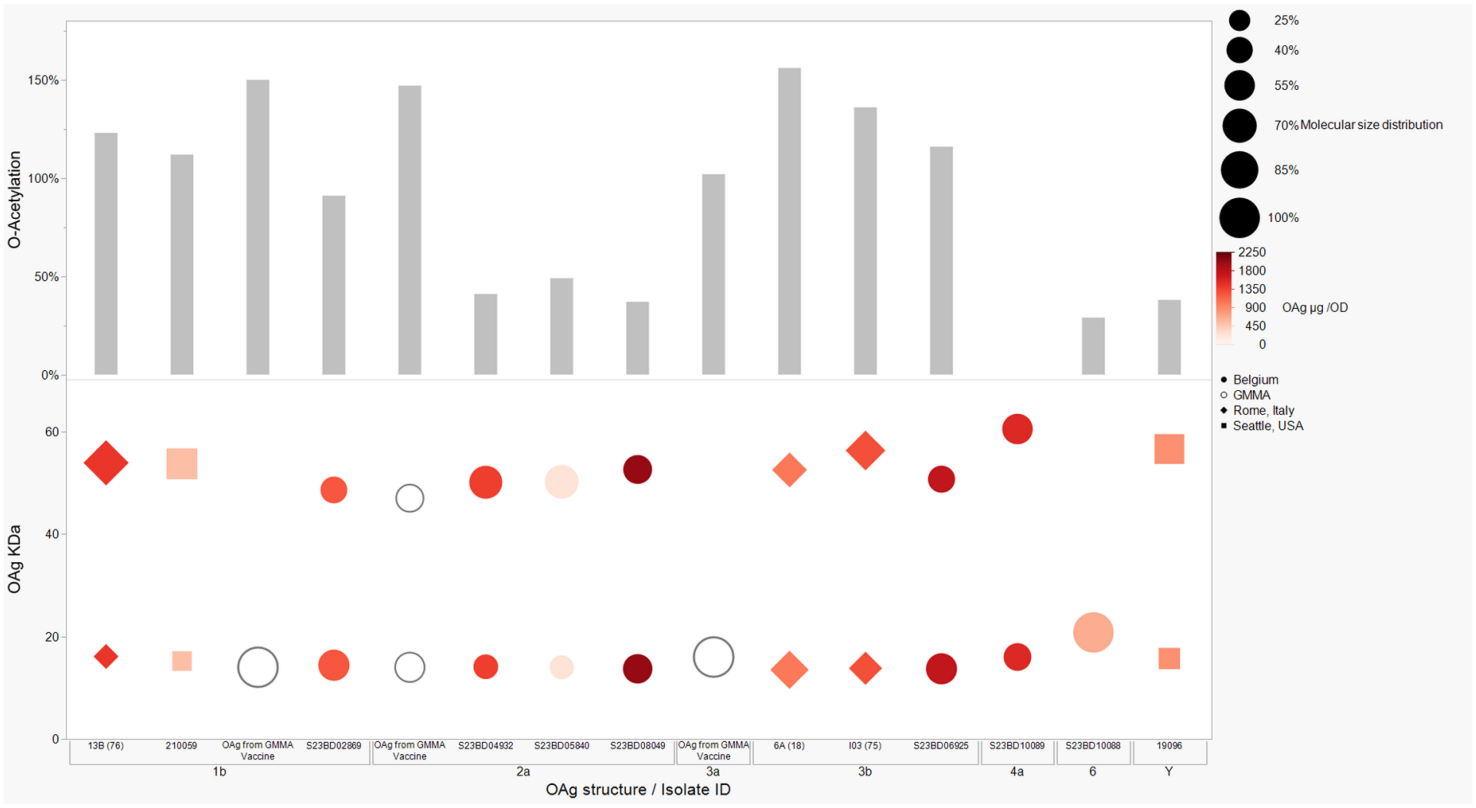
OAg structural features of clinical isolates and GMMA. OAg molecular size distribution of each isolate and *O*-acetylation % are reported on the y-axis: single symbols corresponding to molecular weight (MW) in kDa of each OAg population and OAc % represented by the bars. The OAg peak area population is represented by the different sizes of the symbols. Color codes are associated with the OAg amount normalized per optical density (OD). The different symbols are associated with the provenience of the isolates or GMMA. OAg, O-antigen; GMMA, Generalized Modules for Membrane Antigens.

OAg was extracted from the 12 *S. flexneri* strains and characterized in terms of polysaccharide identity, chain length, quantity, and percentage/position of *O*-acetyl groups (**Table 2**, **Figure 1**). By growing all strains under similar conditions, the amount of OAg produced ranged from 242 µg of OAg per optical density (OD) for the *S. flexneri* 2a strain S23BD05840 (*S. flexneri* 2a) to 2,036 µg of OAg per OD for the *S. flexneri* 2a isolate S23BD08049 (*S. flexneri* 2a). Nuclear magnetic resonance (NMR) spectroscopy was used to confirm the OAg structure of all known serotypes and to identify one of the unknown isolates, comparing the anomeric region of the spectra with previously published data (**Supplementary Figures 1**-**4**) (33). Unknown serotypes were identified as follows: one *S. flexneri* Y (19096), two *S. flexneri* 1b [210059 and 13B(76)], and two *S. flexneri* 3b [I03(75) and 6A(18)]. All isolates except *S. flexneri* 6 S23BD10088 displayed two main OAg populations with different molecular size distributions: a high-molecular-weight (HMW) population at 48.6–60.5 kDa and a medium-molecular-weight (MMW) population at 13.5–16.1 kDa. Interestingly, the *S. flexneri* 6 strain exhibited MMW OAg only, with no peak related to the presence of the capsular polysaccharide detected (34). All isolates, with the exception of *S. flexneri* 4a S23BD10089 (as expected), were *O*-acetylated, and the details on % and positions of *O*-acetyl groups are reported in **Table 2**; **Supplementary Figures 5**-**8**. Interestingly, all *S. flexneri* 3b OAgs presented a low percentage of an additional *O*-acetyl group with respect to the *O*-acetyl on Rha^I^.

**Table 2:** Characterization of O-antigen features in clinical isolates and GMMA composing the altSonflex1-2–3 vaccine.

Isolate ID	OAg structure		OAg quantity	Molecular size distribution kDa and peak area % (HPLC-SEC dRI)		OAc	
	(^1^H-NMR/HPAEC-PAD)		µg/OD			%	Molar ratio
			(HPAEC-PAD)	HMW	MMW	(µHestrin)	(^1^H-NMR)
19096	Y	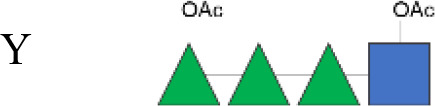	860	56.6 (67%)	15.7 (33%)	38	3.4 (Rha^III^ 4OAc + GlcNAc 6OAc):1 (Rha^III^ 3OAc)
*Shigella flexneri* 1b OAg from GMMA (vaccine)	1b	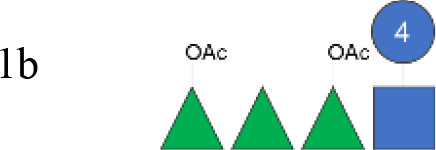	na	nd	14.0 (100%)	150	2.4 (Rha^I^ 2OAc):2.2 (Rha^III^ 3OAc):1 (Rha^III^ 4OAc)
210059	1b	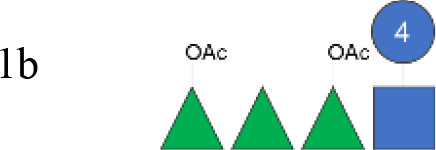	531	53.7 (73%)	15.2 (27%)	112	8.4 (Rha^I^ 2OAc):2.4 (Rha^III^ 3OAc):1 (Rha^III^ 4OAc)
13B (76)	1b	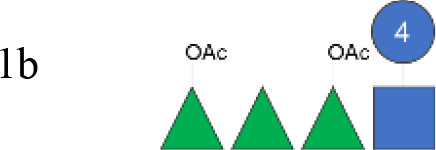	1,445	53.9 (78%)	16.1 (22%)	123	4.1 (Rha^I^ 2OAc):1.1 (Rha^III^ 3OAc):1 (Rha^III^ 4OAc)
S23BD02869	1b	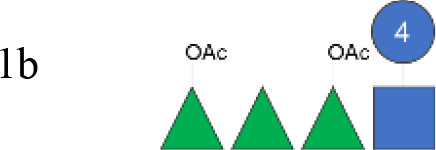	1,241	48.6 (42%)	14.4 (58%)	91	5.7 (Rha^I^ 2OAc):2.3 (Rha^III^ 3OAc):1 (Rha^III^ 4OAc)
*S. flexneri* 2a OAg from GMMA (vaccine)	2a	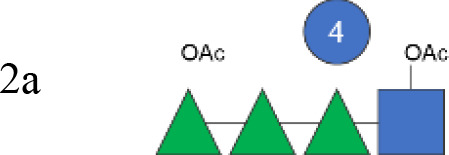	na	47.0 (46%)	14.0 (54%)	147	1.2 (Rha^III^ 4OAc + GlcNAc 6OAc):1 (Rha^III^ 3OAc)
S23BD04932	2a	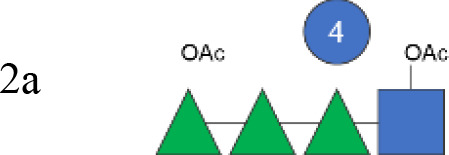	1,390	50.1 (65%)	14.1 (35%)	41	1.6 (Rha^III^ 4OAc + GlcNAc 6OAc):1 (Rha^III^ 3OAc)
S23BD05840	2a	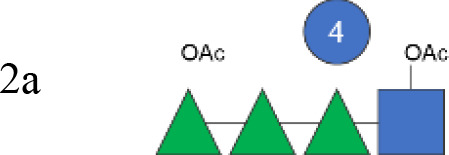	242	50.2 (68%)	14.0 (32%)	49	2.3 (Rha^III^ 4OAc + GlcNAc 6OAc):1 (Rha^III^ 3OAc)
S23BD08049	2a	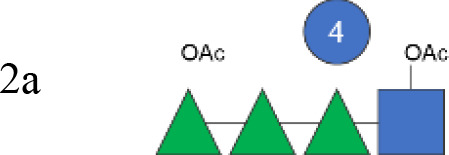	2,036	52.6 (49%)	13.7 (51%)	37	1.9 (Rha^III^ 4OAc + GlcNAc 6OAc):1 (Rha^III^ 3OAc)
*S. flexneri* 3a OAg from GMMA (vaccine)	3a	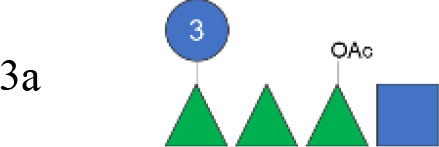	na	nd	16.0 (100%)	102	1 (Rha^I^ 2OAc)
I03 (75)	3b	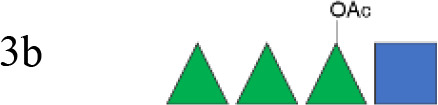	1,282	56.3 (60%)	13.8 (40%)	136	3.8 (Rha^I^ 2OAc):1 (unknown)
6A (18)	3b	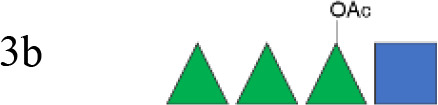	1,020	52.5 (45%)	13.5 (55%)	156	3.3 (Rha^I^ 2OAc):1 (unknown)
S23BD06925	3b	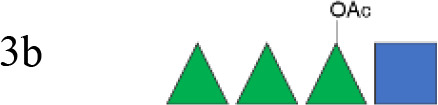	1,760	50.7 (43%)	13.7 (57%)	116	2.8 (Rha^I^ 2OAc):1 (unknown)
S23BD10089	4a	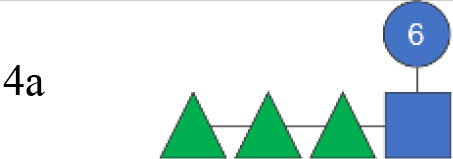	1,551	60.5 (55%)	16.0 (45%)	0	No OAc
S23BD10088	6	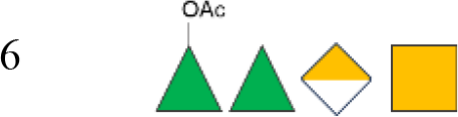	665	nd	20.8 (100%)	29	1.2 (Rha^III^ 3OAc):1 (Rha^III^ 4OAc)

nd, not detected; na, not applicable; GMMA, Generalized Modules for Membrane Antigens; OAg, O-antigen; OD, optical density; HMW, high molecular weight; MMW, medium molecular weight; and HPLC-SEC dRI, high-performance liquid chromatography–size exclusion chromatography with differential refractive index detector.

Isolates expressing the same OAg [*S. flexneri* 1b 210059, 13B(76), and S23BD02869; *S. flexneri* 2a S23BD04932, S23BD05840, and S23BD08049; *S. flexneri* 3b I03(75), 6A(18), and S23BD06925] showed similar structural features, despite producing different amounts of OAg (**Table 2**, **Figure 1**).

To verify possible structural differences, the *S. flexneri* 1b and 2a OAgs extracted from the circulating isolates were compared with the homologous OAg displayed on *S. flexneri* GMMA in the altSonflex1-2–3 vaccine. Interestingly, few differences were observed in terms of both size and *O*-acetylation levels. In particular, the HMW population was not found in the *S. flexneri* 1b OAg displayed by GMMA, which showed the MMW peak only, while both HMW and MMW OAgs were present in *S. flexneri* 2a OAg-GMMA (**Table 2**). Moreover, even if the total *O*-acetylation percentage was similar, all the *S. flexneri* 1b OAgs from the clinical isolates were primarily *O*-acetylated at position 2 of the Rha^I^, while the same OAg on GMMA presented *O*-acetylation also in position 3 of Rha^III^, with a similar ratio between the two positions. All the *S. flexneri* 2a OAgs were instead *O*-acetylated in the same positions with similar ratios, but the overall % of O-acetylation (OAc) was different, with the OAg on GMMA being more *O*-acetylated (147%) compared to clinical strains (37%–49%) (**Table 2**; **Supplementary Figures 5**-**8**).

### Functionality of immune sera against a panel of drug-resistant clinical isolates from recent outbreaks

**Figure 2:**
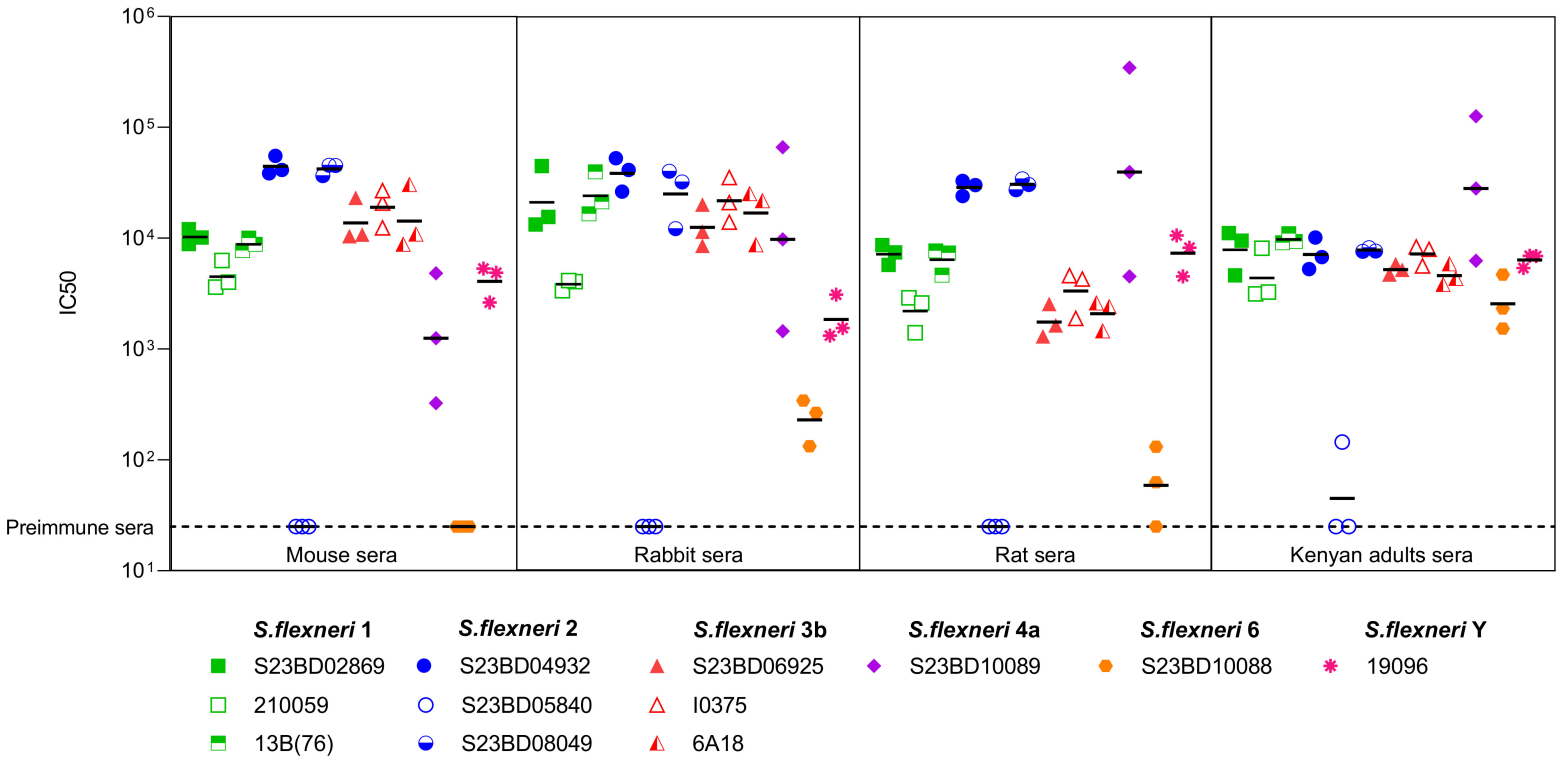
Bactericidal activity of preclinical sera against clinical isolates. Bactericidal activity expressed in IC50 values for each clinical isolate tested with serum of altSonflex1-2-3-immunized mice, rabbits, and rats. IC50 of sera from Kenyan adults from endemic settings is reported for comparison. Three biological replicates for each strain are represented, with central line indicating the geometric mean of the IC50s for each strain.

To prove the potential effectiveness of altSonflex1-2–3 against real-world strains, we tested the bactericidal activity of immune sera derived from three altSonflex1-2-3-vaccinated preclinical models (mice, rabbits, and rats) via luminescence-based serum bactericidal activity (L-SBA) assay (**Figure 2**). As a comparison, a pool of sera from non-vaccinated Kenyan adults, who were considered protected from shigellosis upon repeated exposures throughout their lives, were also tested in L-SBA against the panel of isolates, as a comparison.

All sera were bactericidal against all tested *S. flexneri* 1b strains, confirming vaccine activity against the homologous strain. When tested against *S. flexneri* 2a strains, all sera were bactericidal against all strains except S23BD05840. However, sera from Kenyan adults were also unable to kill this strain, confirming its resistance. As a possible explanation for this observation, strain S23BD05840 was found to be particularly resistant to complement intrinsic toxicity. Also, OAg characterization showed that this strain produced less OAg compared to the other strains. Since LPS is usually considered to protect bacteria from complement deposition, we may hypothesize that the resistance of this strain may be due to other virulence factors.

**Figure 2** also reports bactericidal activity against heterologous strains not included in the vaccine. Importantly, all sera demonstrated bactericidal activity against *S. flexneri* 3b, 4a, and Y strains. Activity against strain 3b can be explained by a very close structural similarity to serotype 3a present in the vaccine. Indeed, the two OAgs only differ in the presence of an additional Glc on Rha^III^ in the RU of *S. flexneri* 3a, compared to *S. flexneri* 3b. Bactericidal activity is expected against *S. flexneri* Y, whose OAg comprises a tetrasaccharide shared by all *S. flexneri* serotypes, with minor *O*-acetylation. Bactericidal activity against *S. flexneri* 4a was also observed, albeit with higher variability compared to that of the other strains. Since this was not observed with 4a laboratory strains (data not shown), we hypothesize this could be related to the extent of growth variability of this specific clinical isolate under the conditions used for SBA. All preclinical sera from vaccinated animals displayed lower or no bactericidal activity against the *S. flexneri* 6 isolate, compared with the sera from Kenyan adults.

For all strains tested, except the *S. flexneri* 6 strain, titers of preclinical sera were similar to those elicited from human sera. In conclusion, our results suggest that altSonflex1-2–3 could offer protection against a panel of homologous and heterologous antimicrobial-resistant *Shigella* strains, with a strong potential impact on global health. altSonflex1-2–3 is being tested in Phase 2 clinical trials (NCT06663436 and NCT05073003), and the functionality of sera from vaccinated infants will be tested against the panel of drug-resistant clinical isolates as well to confirm preclinical data. Testing in the Phase 3 clinical trial will confirm the ability of the vaccine to protect in the field.

## Methods

### Animal sera

GlaxoSmithKline (GSK) is committed to the Replacement, Reduction, and Refinement (3Rs) of animal studies. Non-animal models and alternative technologies are part of our strategy and are employed where possible. When animals are required, the application of robust study design principles and peer review minimizes animal use, reduces harm, and improves benefits in studies. Animal studies were performed in the GSK Animal Facility (Siena, Italy) or at Charles River Laboratories (France), in compliance with the relevant guidelines (Italian D.Lgs. n. 26/14 and European directive 2010/63/UE) and the institutional policies of GSK. The animal protocol approved by the Italian Ministry of Health was AWB project No. 643/2021-PR, with approval date 12/08/2021.

Mouse serum was pooled from 10 female CD1 mice vaccinated intramuscularly (IM) with 50 µL of altSonflex1-2–3 at a dose of 6 μg of total OAg (1.5 μg per serotype) with Alhydrogel. Rabbit serum was pooled from eight female New Zealand White rabbits vaccinated IM with 500 µL of altSonflex1-2–3 at a dose of 60 µg of total OAg (15 μg per serotype) with Alhydrogel. Rat serum was pooled from eight Sprague–Dawley rats vaccinated IM with 200 µL of altSonflex1-2–3 at a dose of 24 µg of OAg (6 µg per serotype) with Alhydrogel. All animals were immunized at study days 0 and 28 and were bled on day 42. All sera were stored frozen in 50 µL working aliquots at −80°C until use. All samples tested in SBA were previously heat-inactivated at 56°C for 30 min to remove endogenous complement activity.

### Clinical sera

The human serum tested was a pool of preimmune sera of 20 Kenyan adults enrolled in the safety cohort of H06_01TP Stage 2 (NCT05073003). All sera were stored frozen in 50 µL working aliquots at −80°C until use. All samples tested in SBA were previously heat-inactivated at 56°C for 30 min to remove endogenous complement activity.

### Bacterial strains

Frozen 20% glycerol stocks were prepared from all *S. flexneri* strains and stored at −80°C until use. To perform experiments, isolates were inoculated from the glycerol stock into a 5-mL Luria-Bertani (LB) liquid culture in preparation for either large-scale growth for OAg extraction or small-scale growth for SBA.

### O-antigen isolation and characterization

A 5-mL LB over-day inoculum of each strain was used to seed a 250-mL LB overnight culture. OD_600nm_ ranged from 1.5 for strain 13B(76) to 8 for strain S23BD05840. OAg was extracted from the bacterial pellet by hydrolysis with 2% acetic acid at 100°C for 4 h and filter-sterilized. The extracted OAgs were purified as previously reported, with slight modifications (35). The samples were subjected to buffer exchange and removal of lower-molecular-mass impurities by ultrafiltration using Amicon Ultra Centrifugal Filter 10 kDa cut-off (Merck, Darmstadt, Germany) against water (3,500 rcf, 10 min, 2 cycles). Next, 25 mM sodium acetate buffer at pH 3.7 was added, reaching a final acetate concentration of 2.5 mM, to precipitate possible protein impurities. After mixing at room temperature (RT) for 30 min, the supernatants were collected through centrifugation and filtered using cation exchange (CEX) chromatography with Sartobind S MA75 filters (Sartorius, Göttingen, Germany) to further remove residual protein impurities. After pH neutralization, the OAg was further purified via anion exchange (AEX) chromatography using a HiTrap Q FF column (Cytiva, Chicago, IL, USA) to remove nucleic acid impurities. All the OAgs eluted at 25 mM NaCl, except the negatively charged *S. flexneri* 6, which eluted at 200 mM NaCl. The resulting solutions were used to calculate the OAg molecular weight using dextrans as standards, as previously reported (36). A final purification step using Amicon Ultra Centrifugal Filter 10 kDa cut-off against water (3,500 rcf, 10 min, 3 cycles) was performed to remove the lipid A core.

OAgs were extracted from GMMA as previously reported (37) and directly used for characterization.

The OAgs were characterized in terms of *O*-acetyl ester content, performing a modified microplate procedure of the Hestrin assay (38), and the OAg sugar composition was estimated by High-Performance Anion-Exchange Chromatography with Pulsed Amperometric Detection (HPAEC-PAD) analysis (36). OAg structures were further confirmed by ^1^H-NMR analysis and measured using a Bruker Avance III 400 spectrometer at 400 MHz and 298 K ^1^H-NMR was also used to determine the position of *O*-acetyl groups.

OAg amounts were quantified by HPAEC-PAD (36) after the first ultrafiltration step (S23BD04932, S23BD05840, S23BD08049, S23BD02869, S23BD06925, S23BD10088, and S23BD10089 OAgs) or after AEX step [19096, 210059, I03(75), 6A(18), and 13B(76) OAg].

### Serum bactericidal assay with luminescence readout

The serum bactericidal assay based on luminescent readout (L-SBA) was conducted as previously described (3, 39, 40). Briefly, sera were serially diluted threefold in Phosphate Buffered Saline (PBS) starting from a 1/50 dilution (25 µL/well). Negative controls without serum were included to monitor non-specific complement killing. Meanwhile, bacteria were cultured to log phase (OD_600nm_ = 0.20 ± 0.02), diluted in the appropriate buffer to 1 × 10^6^ CFU/mL, mixed with baby rabbit complement (BRC) (Cedarlane, Southern Ontario, Canada, CL 3441-S) (**Supplementary Table 1**) to a final volume of 100 μL/well, and incubated at 37°C for 3 h. Bacteria were pelleted and resuspended in 100 μL PBS to remove complement-lysed bacteria. Finally, 25 μL of resuspended live bacteria was mixed with 25 μL of BacTiter-Glo (Promega, Madison, Wisconsin G8231) in a white 384-well plate (Greiner Bio-One, Monroe, North Carolina 655904). The reagents lyse live bacteria, which release ATP and enable bacterial quantification. After 5-min incubation on an orbital shaker at 300 rpm in the dark and RT, ATP-induced luminescence was acquired using a luminometer (Synergy HT, Biotek, Swindon, UK), which correlated with the number of living bacteria in the wells.

Relative luminescence values were plotted in Prism (GraphPad, version 9.5.3) and fitted on the logarithmic value of the serum dilutions tested for each serum in a four-parameter logistic regression model; an arbitrary serum concentration of 10^−15^ was assigned to the well containing no serum (39). To allow correct fitting, the curves were constrained to have a bottom between 0 and the mean of three counts per second corresponding to the lower luminescence detected at T180 in wells where bacteria were killed. The results of the assay are expressed as IC50, representing the dilution of serum able to mediate 50% of killing efficiency with respect to the maximal value. Three independent experiments were performed for each serum on each strain.

The optimal BRC concentration for each strain was established by performing a comprehensive experiment that assessed bacterial growth in a range of increasing BRC percentages in the absence of serum. The selected concentration was determined as the one that most closely resembled the conditions without BRC, wherein the positive impact on bacterial growth was counterbalanced by its inherent toxicity (**Supplementary Table 1**).

## Data Availability

The original contributions presented in the study are included in the article/Supplementary Material, further inquiries can be directed to the corresponding author.
