# Supplementary material for: Antibodies elicited by altSonflex1-2-3 GMMA vaccine are bactericidal against a panel of drug-resistant Shigella clinical isolates
Source: Front Immunol. 2025 Sep 4;16:1652460. doi: 10.3389/fimmu.2025.1652460 (PMC12443847; doi:10.3389/fimmu.2025.1652460)
Supplement: Supplementary file 1 [file DataSheet1.docx]

**Supplementary information**

**Supplementary Table 1. SBA conditions per strain.**

| **Strain** | **Assay buffer** | **BRC percentage** |
| --- | --- | --- |
| 19096 | Luria Broth | 15 |
| 210059 | Luria Broth | 15 |
| 1AI03 | Luria Broth | 25 |
| 6A18 | Luria Broth | 25 |
| 13B76 | Luria Broth | 8 |
| S23BD04932 | Luria Broth | 15 |
| S23BD05840 | Luria Broth | 30 |
| S23BD08049 | Luria Broth | 15 |
| S23BD02869 | Luria Broth | 15 |
| S23BD06925 | Luria Broth | 20 |
| S23BD10088 | Luria Broth | 20 |
| S23BD10089 | Luria Broth | 15 |

**Supplementary Figures**

**
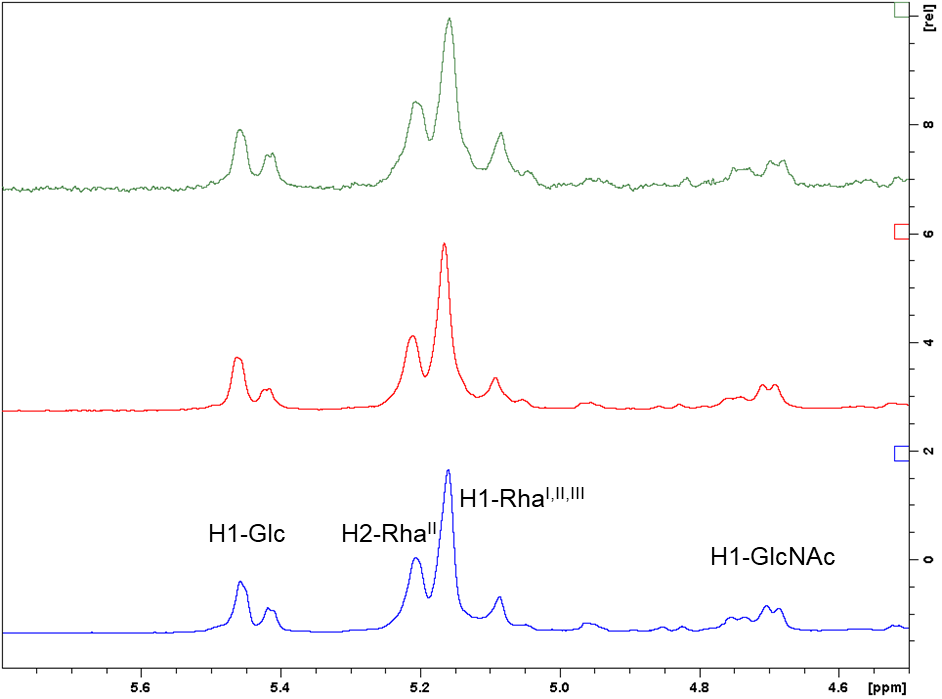
**

**Supplementary Figure 1.** ^1^H NMR spectra with zoom on the anomeric region of OAg extracted from *S. flexneri* 1b strains 210059 (blue line), 13B (76) (red line) and S23BD02869 (green line). Some assignments are labelled based on[^1^](#_ENREF_1).

**
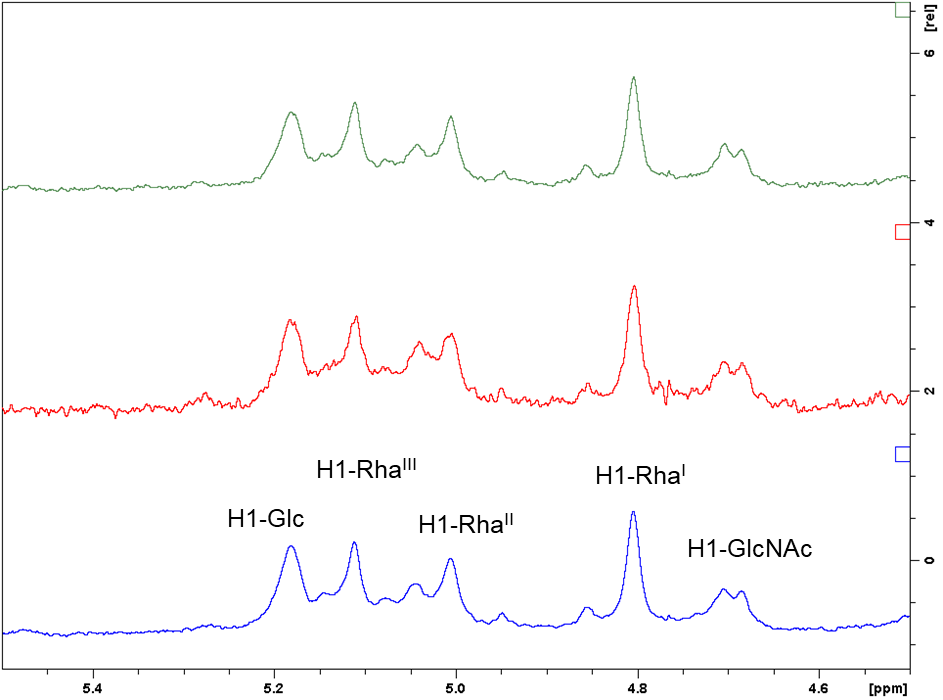
**

**Supplementary Figure 2.** ^1^H NMR spectra with zoom on the anomeric region of OAg extracted from *S. flexneri* 2a strains S23BD04932 (blue line), S23BD05840 (red line) and S23BD08049 (green line). Some assignments are labelled based on^1^.

**
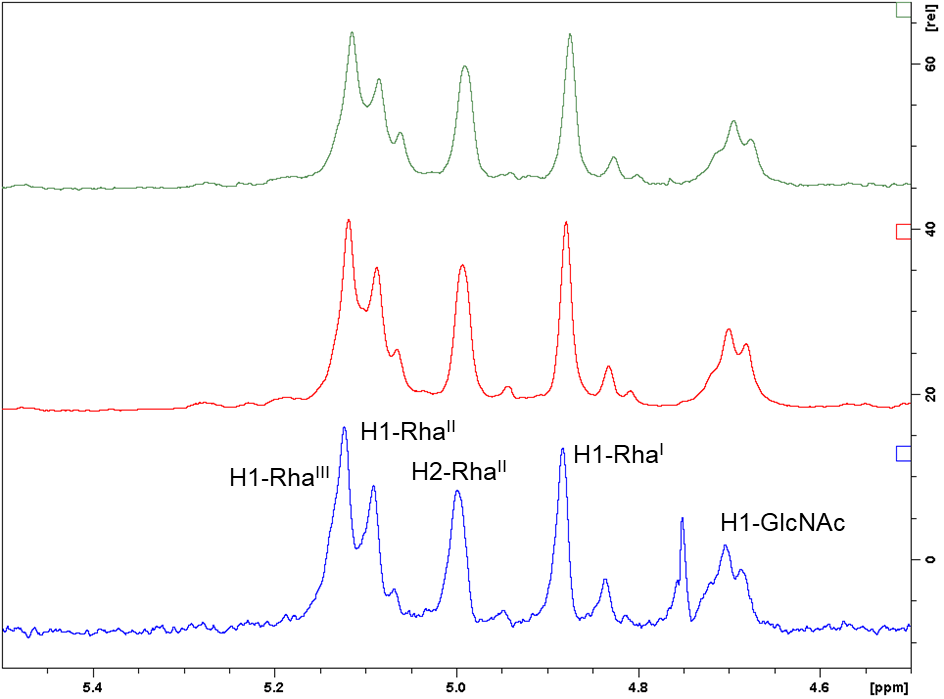
**

**Supplementary Figure 3.** ^1^H NMR spectra with zoom on the anomeric region of OAg extracted from *S. flexneri* 3b strains I03 (75) (blue line), 6A (18) (red line) and S23BD06925 (green line). Some assignments are labelled based on^2^.

**
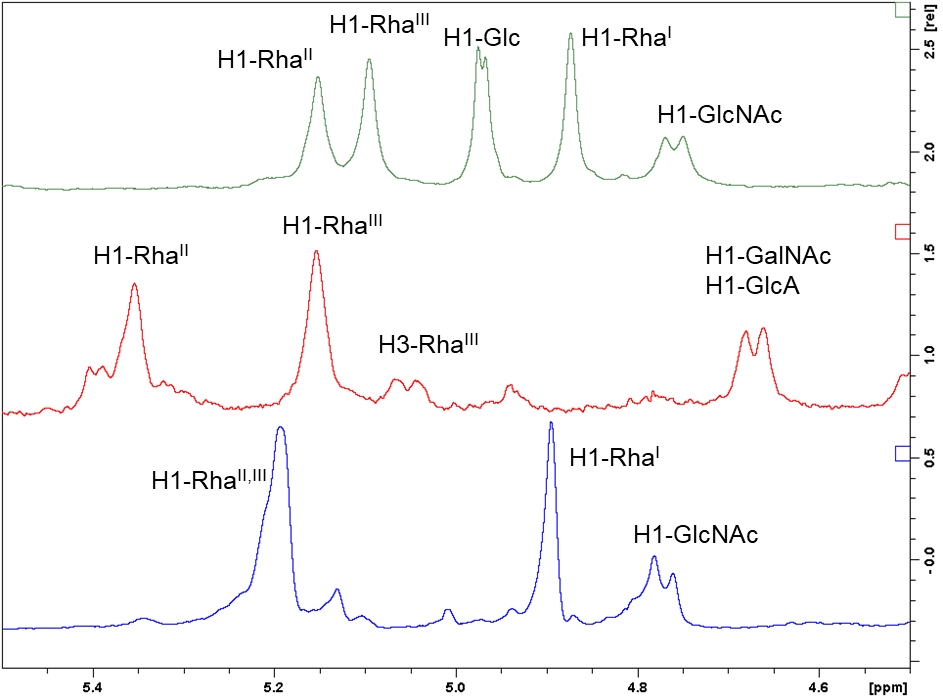
**

**Supplementary Figure 4.** ^1^H NMR spectra with zoom on the anomeric region of OAg extracted from *S. flexneri* Y strain 19096 (blue line), *S. flexneri* 6 strain S23BD10088 (red line) and *S. flexneri* 4a strain S23BD10089 (green line). Some assignments are labelled based on^2^.


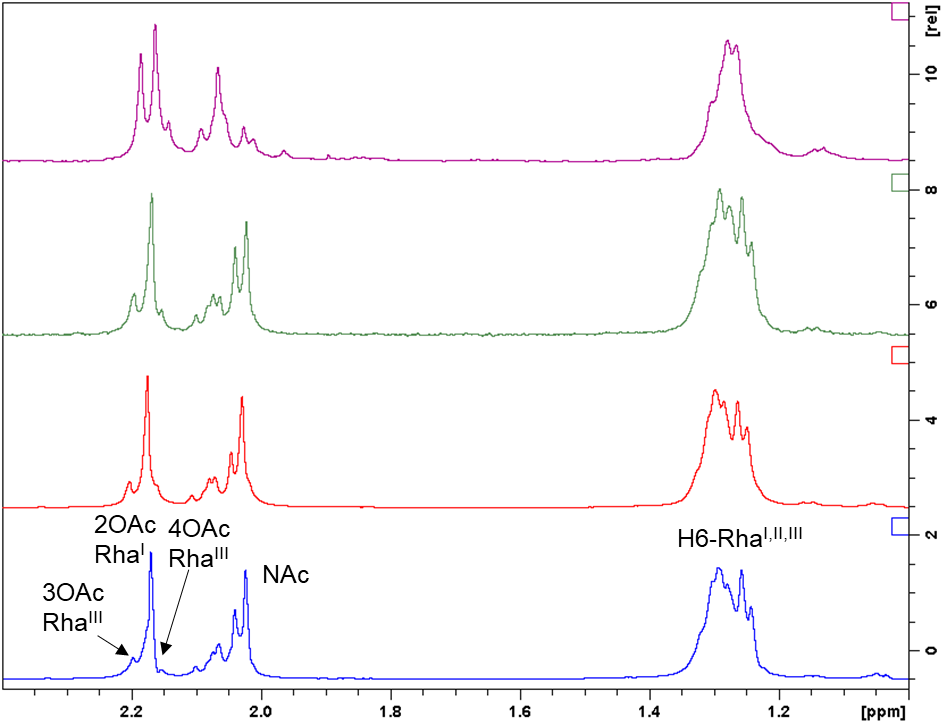


**Supplementary Figure 5.** ^1^H NMR spectra with zoom on the O-Acetyl region of OAg extracted from *S. flexneri* 1b strains 210059 (blue line), 13B (76) (red line), S23BD02869 (green line) and from *S. flexneri* 1b GMMA (violet line). Some assignments are labelled based on^1^.


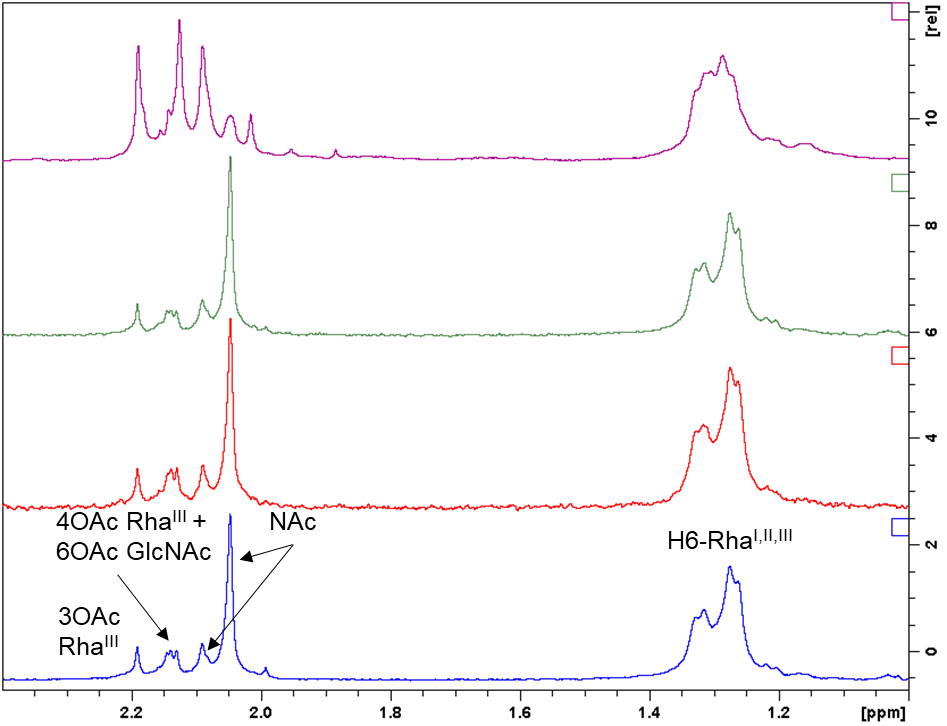


**Supplementary Figure 6.** ^1^H NMR spectra with zoom on the O-Acetyl region of OAg extracted from *S. flexneri* 2a strains S23BD04932 (blue line), S23BD05840 (red line), S23BD08049 (green line) and from *S. flexneri* 2a GMMA (violet line). Some assignments are labelled based on^1^.


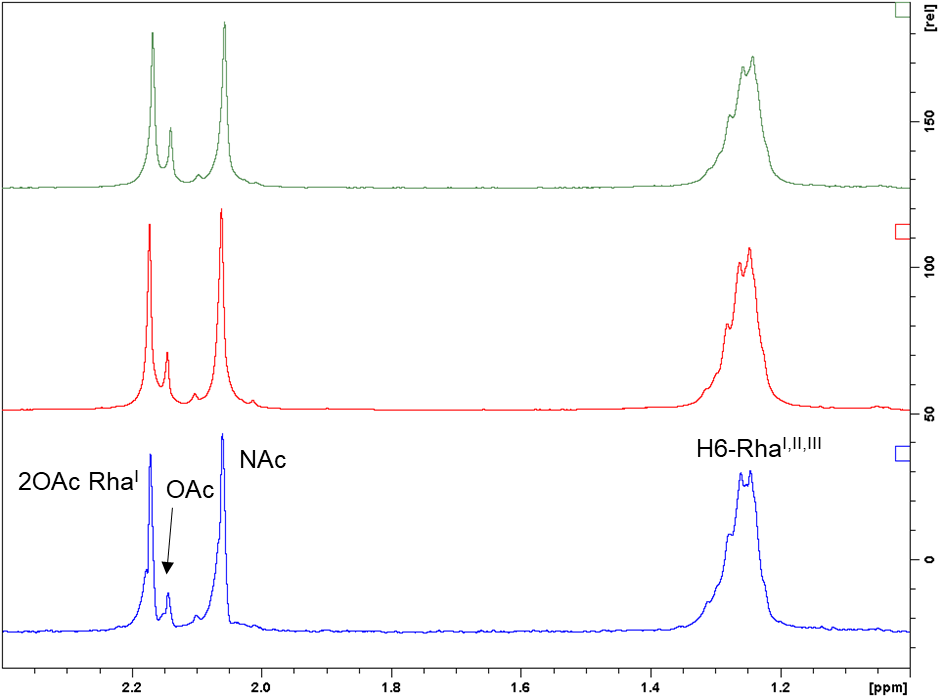


**Supplementary Figure 7.** ^1^H NMR spectra with zoom on the O-Acetyl region of OAg extracted from *S. flexneri* 3b strains I03 (75) (blue line), 6A (18) (red line) and S23BD06925 (green line). Some assignments are labelled based on^2^.

**
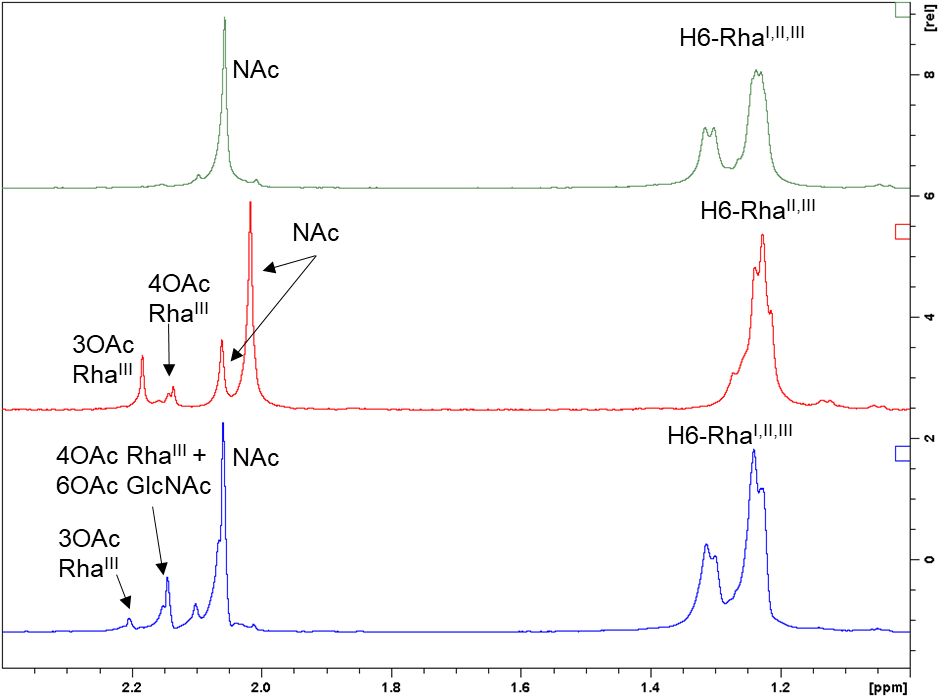
**

**Supplementary Figure 8.** ^1^H NMR spectra with zoom on the O-Acetyl region of OAg extracted from *S. flexneri* Y strain 19096 (blue line), *S. flexneri* 6 strain S23BD10088 (red line) and *S. flexneri* 4a strain S23BD10089 (green line). Some assignments are labelled based on^2^.

1 Perepelov, A. V. *et al.* A similarity in the O-acetylation pattern of the O-antigens of Shigellaflexneri types 1a, 1b, and 2a. *Carbohydr Res* **344**, 687-692 (2009). <https://doi.org/10.1016/j.carres.2009.01.004>

2 Jansson, P.-E., Kenne, L. & Wehler, T. A 2D-1H-N.M.R. study of some Shigella flexneri O-polysaccharides. *Carbohydrate Research* **166**, 271-282 (1987). <https://doi.org/https://doi.org/10.1016/0008-6215(87)80063-0>
